# Administration of umbilical cord mesenchymal stem cells in patients with severe COVID-19 pneumonia

**DOI:** 10.1186/s13054-020-03142-8

**Published:** 2020-07-11

**Authors:** Zhinian Guo, Yunlong Chen, Xiaoyu Luo, Xiaolong He, Yong Zhang, Jiang Wang

**Affiliations:** Department of Cardiology, Xinqiao Hospital of Army Medical University, 183 Xinqiao Street, Chongqing, 400037 China

There are no specific drug therapies or vaccines for the pandemic of coronavirus disease 2019 (COVID-19), which is associated with substantial mortality. Attenuating or reversing the cytokine storm is critical for treating patients with severe COVID-19 pneumonia. Mesenchymal stem cells (MSCs) have been shown to have powerful immunoregulation and reparative properties in injured tissue with good safety [1]. This report aims to investigate whether umbilical cord MSC (UC-MSC) therapy improves the outcomes of 31 patients with severe or critical COVID-19 pneumonia.

We wish to report our experience using UC-MSCs for the treatment of severe COVID-19 pneumonia at Taikangtongji Hospital in Wuhan, China, from January 3, 2020, to April 4, 2020. Patient data, including demographics, clinical data, laboratory indices, treatment, and in-hospital outcomes, were collected. Severe acute respiratory syndrome coronavirus 2 (SARS-CoV-2) polymerase chain reaction (PCR) results of all patients were positive before UC-MSCs infused. Patients were diagnosed and treated according to national guidelines. Before the intravenous drip was established, UC-MSCs (1 × 10^6^ cells per kilogram of weight) were suspended in 100 ml normal saline. We report numbers (percentages) for categorical variables and the median (interquartile range [IQR]) or mean ± standard deviation (SD) for continuous variables. Intergroup comparisons were performed with paired *t* tests.

We treated 31 COVID-19 patients with UC-MSCs. The median age was 70 years (IQR, 61–71 years); 25 patients (80.6%) were male. The proportion of treatment with oxygen was the highest (31 [100%]), followed by antivirals (26 [83.9%]), antibiotics (23 [74.2%]), intravenous immunoglobulin (8 [25.8%]), intravenous albumin (8 [25.8%]), and methylprednisolone (6 [19.4%]). The median (IQR) volume of infused UC-MSCs was 200 mL (100–300 mL). No adverse events were attributable to intravenous transplantation of UC-MSCs. After the first infusion of UC-MSCs, the SARS-CoV-2 PCR results of 30 patients (96.8%) became negative after a mean time of 10.7 days (SD, 4.2 days) (Table 1). Laboratory parameters tended to improve after UC-MSC therapy compared to the status before UC-MSC therapy, including elevated lymphocyte count (median [IQR], 1.09 [0.68–1.35] × 10^9^/L vs 1.43 [1.02–2.20] × 10^9^/L; *P* <  0.001), decreased C-reactive protein level (median [IQR], 13.39 [1.30–38.86] mg/L vs 0.50 [0.50–6.40] mg/L; *P* = 0.003), decreased procalcitonin level (median [IQR], 0.07 [0.05–0.09] ng/mL vs 0.04 [0.03–0.06] ng/mL; *P* <  0.001), decreased interleukin-6 level (median [IQR], 13.78 [5.69–25.26] pg/mL vs 4.86 [2.13–8.19] pg/mL; *P* <  0.001), decreased D-dimer level (median [IQR], 495 [320–727] ng/mL vs 288 [197–537] ng/mL; *P* = 0.010), and elevated PaO_2_/FiO_2_ (median [IQR], 242 [200–294] vs 332 [288–364]; *P* <  0.001) (Table 2).

**Table 1:** Baseline characteristics, treatments, and outcomes of patients with COVID-19 with UC-MSC therapy

	Total (*n* = 31)
Demographics and clinical characteristics	
Age, median (IQR), years	70 (61–71)
Sex, male	25 (80.6%)
BMI, mean ± SD, kg/m^2^	24.5 ± 2.9
Symptoms at admission	
Fever	24 (77.4%)
Cough	25 (80.6%)
Dyspnea	17 (54.8%)
Chest congestion	14 (45.2%)
Fatigue	12 (38.7%)
Comorbidities	
Hypertension	13 (41.9%)
Chronic obstructive pulmonary disease	6 (19.4%)
Coronary artery disease	5 (16.1%)
Diabetes	5 (16.1%)
Chest computed tomographic findings	
Bilateral pneumonia	31 (100%)
Multiple mottling/ground-glass opacity	26 (83.9%)
Main complications	
Respiratory failure	10 (32.3%)
Acute respiratory distress syndrome	8 (25.8%)
Cardiac injury	12 (38.7%)
Disease severity status	
Severe	23 (74.2%)
Critical	8 (25.8%)
Days between onset of symptoms and hospital admission, mean ± SD, days	37.2 ± 17.6
Days between onset of symptoms and UC-MSC therapy, mean ± SD, days	50.7 ± 12.6
Days between hospital admission and UC-MSC therapy, median (IQR), days	10.0 (6.0–22.0)
Intensive care unit admission	16 (51.6%)
Treatments	
Oxygen	31 (100%)
Oxygen inhalation	19 (61.3%)
Noninvasive mechanical ventilation	4 (12.9%)
Invasive mechanical ventilation	8 (25.8%)
Antivirals	26 (83.9%)
Arbidol	20 (64.5%)
Interferon alfa-2b	9 (29.0%)
Oseltamivir	3 (9.7%)
Chloroquine	3 (9.7%)
Antibiotics	23 (74.2%)
Methylprednisolone	6 (19.4%)
UC-MSC therapy	
UC-MSC volume, median (IQR), mL	200 (100–300)
Single infusion of UC-MSCs	11 (35.5%)
Two infusions of UC-MSCs	9 (29.0%)
Three infusions of UC-MSCs	11 (35.5%)
Intravenous immunoglobulin therapy	8 (25.8%)
Intravenous albumin therapy	8 (25.8%)
Outcomes	
SARS-CoV-2 clearance	30 (96.8%)
Discharged	27 (87.1%)
Death	4 (12.9%)

*UC-MSCs* umbilical cord mesenchymal stem cells, *IQR* interquartile range, *SD* standard deviation, *SARS-CoV-2* severe acute respiratory syndrome coronavirus 2

**Table 2:** Comparison of laboratory parameters before and after UC-MSC therapy

Characteristics	Before UC-MSC therapy	After UC-MSC therapy	*P* value
White blood cell count, × 10^9^/mL (normal range, 3.5–9.5)	6.72 ± 2.62	6.43 ± 1.72	0.346
Lymphocyte count, × 10^9^/mL (normal range, 1.1–3.2)	1.09 (0.68–1.35)	1.43 (1.02–2.20)	**< 0.001**
C-reactive protein, mg/L (normal range, < 10)	13.39 (1.30–38.86)	0.50 (0.50–6.40)	**0.003**
Procalcitonin, ng/mL (normal range, < 0.05)	0.07 (0.05–0.09)	0.04 (0.03–0.06)	**< 0.001**
Interleukin-6, pg/mL (normal range, < 7)	13.78 (5.69–25.26)	4.86 (2.13–8.19)	**< .001**
D-dimer, ng/mL (normal range, < 243)	495 (320–727)	288 (197–537)	**0.010**
PaO_2_/FiO_2_	242 (200–294)	332 (288–364)	**< 0.001**

*UC-MSCs* umbilical cord mesenchymal stem cells, *PaO*_*2*_*/FiO*_*2*_*ratio* ratio of the partial pressure of arterial oxygen to the percentage of inspired oxygen

Our experience showed that UC-MSC therapy may restore oxygenation and downregulate cytokine storms in patients hospitalized with severe COVID-19 without any infusion reaction. This approach is a promising candidate for the treatment of severe COVID-19 [2]. During the outbreak of COVID-19 in Wuhan, China, the number of patients increased sharply. However, the hospital capacity was limited, and many patients could not be admitted to the hospital. Hence, days between onset of symptoms and hospital admission were long. UC-MSCs can improve the lung microenvironment, pulmonary fibrosis, and lung function, probably due to the regulation of the inflammatory response and the promotion of tissue repair and regeneration [3]. A recent report of 7 patients found that bone marrow MSC therapy was an effective treatment for severe COVID-19 [3]. Moreover, another recent study indicated that bone marrow MSC therapy can improve hypoxia, immune reconstitution, and cytokine storms in patients with severe COVID-19 [4], which was consistent with our results. Further large multiple-center prospective trials are needed to confirm our results in the future.

## Data Availability

The data used to support the findings of this study are available from the corresponding author upon request.
